# Enrichment of HLA Types and Single-Nucleotide Polymorphism Associated With Non-progression in a Strictly Defined Cohort of HIV-1 Controllers

**DOI:** 10.3389/fimmu.2017.00746

**Published:** 2017-06-27

**Authors:** Samantha J. Westrop, Alexander T. H. Cocker, Adriano Boasso, Ann K. Sullivan, Mark R. Nelson, Nesrina Imami

**Affiliations:** ^1^Centre for Immunology and Vaccinology, Imperial College London, London, United Kingdom; ^2^Department of HIV/GU Medicine, Chelsea and Westminster Hospital, London, United Kingdom

**Keywords:** HIV-1, disease progression, elite controllers, HLA antigens, single-nucleotide polymorphism

## Abstract

HIV-1 controllers (HIC) are extremely rare patients with the ability to control viral replication, maintain unchanging CD4 T-cell count, and evade disease progression for extensive periods of time, in the absence of antiretroviral therapy. In order to establish the representation of key genetic correlates of atypical disease progression within a cohort of HIV-1^+^ individuals who control viral replication, we examine four-digit resolution HLA type and single-nucleotide polymorphisms (SNP) previously identified to be correlated to non-progressive infection, in strictly defined HIC. Clinical histories were examined to identify patients exhibiting HIC status. Genomic DNA was extracted, and high definition HLA typing and genome-wide SNP analysis was performed. Data were compared with frequencies of SNP in European long-term non-progressors (LTNP) and primary infection cohorts. HLA-B alleles associated with atypical disease progression were at very high frequencies in the group of five HIC studied. All four HIC of European ancestry were HLA-B*57^+^ and half were also HLA-B*27^+^. All HIC, including one of self-reported African ethnicity, had the HLA-Cw*0602 allele, and the HLA-DQ9 allele was present only in HIC of European ancestry. A median 95% of the top 19 SNP known to be associated with LTNP status was observed in European HIC (range 78–100%); 17/19 of the SNP considered mapped to chromosome 6 in the HLA region, whereas 2/19 mapped to chromosome 8. The HIC investigated here demonstrated high enrichment of HLA types and SNP previously associated with long-term non-progression. These findings suggest that the extreme non-progressive phenotype considered here is associated with a genetic signature characterized by a single-genetic unit centered around the HLA-B*57 haplotype and the possible additive effect of HLA-B*27.

## Introduction

A very small proportion of over 7,000 HIV-1^+^ patients, currently attending the Chelsea and Westminster Hospital have been identified by our group as HIV-1 controllers (HIC) (1, 2). These individuals meet the following strictly defined criteria: (i) infected with HIV-1 for ≥7 years, (ii) maintain stable CD4^+^ T-cell counts within the normal healthy range (450–1,650 cells/μl blood; slope ≥0 cells/μl blood) throughout clinical follow up, (iii) suppress HIV-1 plasma RNA levels to below detectable limit (<50 copies/ml plasma), and (iv) no history of opportunistic infection despite never receiving antiretroviral therapy (1–3). These unusual patients provide an opportunity to establish objectives for immunotherapy in HIV-1^+^ individuals, who exhibit irreversible decline of immune function, despite otherwise successful suppressive antiretroviral therapy (4).

HLA types have long been associated with varying rates of disease progression (5), and differing effects have been reported between subtypes of HLA alleles (6), indicating that HLA typing to a high resolution (i.e., four digits) that defines the antigen-binding site is required to provide relevant distinguishing information. This is particularly important as specificity at the amino acid level within the MHC class I molecule-binding groove affects peptide presentation and is a major determinant of clinical phenotype (7, 8).

Genome-wide association studies (GWAS) enable the exploration of various genetic factors on HIV-1 susceptibility, control, and pathogenesis (9). GWAS allow investigation of single-nucleotide polymorphism (SNP) profiles of interesting individuals. Furthermore, genetic correlates of phenotype can be deciphered through comparison with a control group (10). We previously performed a GWAS on a cohort of individuals defined as long-term non-progressors (LTNP) (11). However, plasma viral load was not specified in the inclusion criteria for this LTNP cohort, and the HLA class I and II types of the patients had not been studied in this main GWAS study.

Here, we report a substantial enrichment of both HLA types and SNP, previously identified as associated with non-progression, in the group of rare HIC identified in the Chelsea and Westminster Hospital cohort using consistently undetectable viral load of <50 copies/ml plasma at every visit over the >7 year period follow-up as one of the inclusion criteria.

## Materials and Methods

Five patients who formed a subset of participants studied in a larger GWAS were identified within the Chelsea and Westminster Hospital cohort who met the HIC criteria previously defined (1, 2). The group of five HIC had a median CD4 T-cell count 882 cells/μl blood (IQR: 688–985), from a total of 74 visits over a period of 72.7 patient years of follow-up. The CD4 T-cell count slopes for each of these individuals were not significantly different from 0 (i.e., non-declining) over the entire period of clinical follow-up. All individuals investigated were male, clade B infected, with a median age of 42 years (IQR 40–45). Of the five HIC, one was of self-reported African ethnicity, while the other four were European Caucasian. Following ethical approval from the National Research Ethics Committee, written informed consent was obtained prior to blood sample collection. Genomic DNA was isolated as previously described (2). HLA typing at four-digit resolution was performed (HLA class I by reference strand conformation analysis and class II by single-specific primer PCR; Department of Clinical Immunology, Histocompatibility and Immunogenetics Laboratory, Hammersmith Hospital). GWAS was carried out as part of the previously described collaborative study (11). For each HIC, high definition HLA type, ethnicity, and length of time since first diagnosis as HIV-1^+^, are detailed in Table 1.

**Table 1:** Patient characteristics and the high definition HLA types for the HIV-1 controllers (HIC) investigated.

	S691	M431	O777	W430	B015
Cohort	HIC	HIC	HIC	HIC	HIC
Self-reported ethnicity	Caucasian	Caucasian	Caucasian	Caucasian	African
Age at time of sample, years	67	42	40	40	45
Time since HIV-1^+^ diagnosis, years	22.9	12.0	7.8	7.8	22.3
Median CD4 T-cell count over time, cells/μl blood	654	856	745	1,035	932
Nadir CD4 T-cell count, cells/μl blood	490	760	518	986	588
HLA-A	0201/09	0101	0101	2402/09	3402
	0301	1101	6802	1101	6802
HLA-B	2702^a^	2705^a^	1402	4402	8101
	5701^a^	5701^a^	5701^a^	5701^a^	3501/2^a^
HLA-Bw	4	4	4	4	6
			6		
HLA-Cw	0202	0202	0602^a^	0501	1801
	0602^a^	0602^a^	0802/04	0602^a^	0602^a^
HLA-DR	0901	0103	0701	0701	0804
	1401	07	1303	1301	1301
	52	53	52	52	52
	53		53	53	
HLA-DQ	5	5	7	6	4
	9	9	9	9	6

*^a^HLA alleles associated with differential rates of disease progression (12–14)*.

## Results

HLA typing revealed a high representation of HLA-B*5701 (100% of Caucasian HIC; 4/4; Table 1), found to be 38.5 times more frequent in these four Caucasian HIC than in a control group of 9,510 Europeans (www.ncbi.nlm.nih.gov/projects/gv/mhc). Two HIC were HLA-B*27^+^ (Table 1). The unusual HLA-B*2702 allele, found to be present in one of four Caucasian HIC, was found at a very low frequency of 0.002 in the European control group. The more frequent B*2705 allele (*f*_[European control group]_ = 0.016), was also found to be present in one of the four Caucasian HIC. HLA-Cw*0602 was present in 100% of the HIC investigated here, whereas the allelic frequency in the European population is 0.091 (*n* = 293; www.ncbi.nlm.nih.gov/projects/gv/mhc). All four Caucasian HIC in this study group had the HLA-DQ9 allele.

Genome-wide association studies were performed on the HIC included in this study as part of the collaborative GISHEAL study, along with 139 others who fulfilled LTNP criteria from the French asymptomatic long term, Italian evaluation of LTNP viro-immunologic study (ELVIS), and additional patients from the Chelsea and Westminster cohort (11, 15, 16), in which viral loads were not taken into account. The group of HIC described here, however, was stringently defined using undetectable viral load (<50 copies/ml plasma at every visit over the >7 year period follow up) as one of the inclusion criteria. SNP of interest, recently shown to correlate with non-progressive HIV-1 infection (11), were identified by comparing profiles of atypical progressors with the control French PRIMO cohort (11, 17). We focused our analysis on the selected SNP that were significantly over—or under—represented in the LTNP cohort. The minor allele of these SNP occurred at increased frequency in the five HIC investigated (Table 2). The four Caucasian HIC carried the minor allele (either homozygous or heterozygous) in 78–100% (median 95%) of the top 19 SNP over-represented in LTNP (11). Conversely, the minor alleles for the five SNP under-represented in LTNP were observed only in 0–60% (median 20%) of the four Caucasian HIC (Table 2). The median frequency of the minor allele in the top 19 over-represented SNP was 0.50 (range 0.25–0.88) among all five HIC investigated, with 80% (range 40–100) of HIC carrying the minor allele in the SNP of interest. When the HIC of African ancestry was excluded and the four Caucasian HIC were analyzed, the median allelic frequency of the top 19 over-represented SNP increased to 0.63 (range 0.33–0.88), with 100% (range 67–100) of HIC carrying the minor allele in the SNP of interest (Table 2).

**Table 2:** Representation of single-nucleotide polymorphism (SNP) found at a significantly higher or lower frequency in the GISHEAL long-term non-progressors (LTNP) cohort (11), in the five atypical progressors.

SNP name	Chr	Position	Gene	Minor allele	Rep. in LTNP^a^	S691	M431	O777	W430	B015	MAF^b^ [without B015]	% with SNP [without B015]	AF in GISHEAL LTNP (11)	AF in PRIMO Cohort (11)
rs1051794	6	31487088	Intra. MICA	A	+	**A**/**A**	**A**/**A**	**A**/**A**	**A**/G	**A**/G	0.80 [0.88]	100 [100]	0.48	0.31
rs2248372	6	31554445	Inter. HCP5/MICB	A	+	**A**/**A**	**A**/**A**	**A**/G	**A**/G	**A**/G	0.70 [0.75]	100 [100]	0.50	0.35
rs2248462	6	31554775	Intra. HLA-B	A	+	**A**/G	**A**/**A**	**A**/G	**A**/G	G/G	0.50 [0.63]	80 [100]	0.38	0.23
rs2395029	6	31539759	Intra. HCP5	G	+	T/**G**	T/**G**	T/**G**	T/**G**	T/T	0.40 [0.50]	80 [100]	0.12	0.03
rs2516509	6	31557973	Inter. HCP5/MICB	G	+	x/x	**G**/**G**	A/**G**	A/**G**	A/A	0.50 [0.67]	75 [100]	0.38	0.23
rs2523619	6	31426123	Inter. HLA-B/MICA	G	+	A/**G**	A/**G**	A/**G**	A/**G**	A/A	0.40 [0.50]	80 [100]	0.34	0.20
rs2894207	6	31371730	Intra. HLA-B	C	+	T/**C**	T/**C**	T/**C**	T/**C**	T/T	0.40 [0.50]	80 [100]	0.31	0.18
rs4711269	6	31462798	Inter. HLA-B/MICA	T	+	**T**/**T**	**T**/**T**	**T**/**T**	**T**/C	**T**/C	0.80 [0.88]	100 [100]	0.46	0.27
rs7772549	6	31515622	Inter. MICA/HCP5	C	+	**C**/**C**	**C**/**C**	**C**/**C**	T/**C**	x/x	0.88 [0.88]	100 [100]	0.48	0.32
rs9266825	6	31490861	Intra. MICA	A	+	**A**/**A**	**A**/**A**	**A**/C	**A**/C	C/C	0.60 [0.75]	80 [100]	0.41	0.28
rs9368699	6	31910520	Inter. upstream C6orf48	C	+	T/T	T/**C**	T/**C**	T/**C**	T/T	0.30 [0.38]	60 [75]	0.13	0.03
rs9378127	6	33030437	Inter. HLA-DMA/BRD2	A	+	G/G	x/x	**A**/G	**A**/G	G/G	0.25 [0.33]	40 [67]	0.22	0.11
rs9469003	6	31515807	Inter. MICA/HCP5	C	+	T/**C**	T/**C**	T/**C**	T/**C**	T/T	0.40 [0.50]	80 [100]	0.27	0.15
rs10484554	6	31382534	Inter. MICA/HCP5	T	+	**T**/C	x/x	x/x	x/x	C/C	0.25 [0.50]	50 [100]	0.25	0.13
rs12198173	6	32134786	Inter. TNXB	A	+	G/G	**A**/G	**A**/**A**	**A**/G	**A**/G	0.50 [0.50]	80 [75]	0.17	0.08
rs13199524	6	32174743	Intra. TNXB	T	+	C/C	x/x	**T**/**T**	**T**/C	C/C	0.38 [0.50]	50 [75]	0.16	0.08
rs13437082	6	31462539	Inter. HLA-B/MICA	T	+	**T**/**T**	**T**/**T**	**T**/**T**	**T**/C	**T**/C	0.80 [0.88]	100 [100]	0.46	0.27
rs7008604	8	125051396	Intra. FER1L6	G	+	**G**/**G**	A/**G**	A/A	**G**/**G**	A/A	0.50 [0.63]	60 [75]	0.47	0.62
rs11993947	8	125040636	Intra. FER1L6	T	+	**T**/**T**	**T**/G	G/G	**T**/**T**	G/G	0.50 [0.63]	60 [75]	0.48	0.62
			No. of loci with SNP (%)			14/18 (78)	16/16 (100)	16/18 (89)	18/18 (100)	5/18 (28)				
rs2523535	6	31444229	Inter. HLA-B/MICA	C	−	T/T	T/T	T/T	T/**C**	T/T	0.10 [0.13]	20 [25]	0.22	0.38
rs2844511	6	31497763	Inter. HCP5/MICB	T	−	C/C	C/C	C/C	**T**/C	**T**/C	0.20 [0.13]	40 [25]	0.19	0.38
rs2844513	6	31496193	Inter. MICA/HCP5	C	−	T/T	T/T	T/T	T/**C**	T/**C**	0.20 [0.13]	40 [25]	0.30	0.48
rs3130805	6	29414435	Inter. OR14J1/OR5V1	A	−	G/G	**A**/G	G/G	G/G	G/G	0.10 [0.13]	20 [25]	0.03	0.10
rs6930435	6	29409201	Inter. OR14J1/OR5V1	A	−	G/G	**A**/G	G/G	G/G	G/G	0.10 [0.13]	20 [25]	0.12	0.20
			No. of loci with SNP (%)			0/5 (0)	2/5 (40)	0/5 (0)	3/5 (60)	2/5 (40)				

*^a^Representation of allele in GISHEAL cohort of LTNP compared to PRIMO cohort*.

*+, increased frequency; −, decreased frequency; AF, allele frequency; MAF, minor allele frequency; x, data not available*.

*^b^Calculated from the group of five atypical progressors*.

*SNP defined as minor in the GISHEAL study of LTNP are in bold*.

Of note, 17 of the 19 SNP considered map to chromosome 6 within the HLA region (i.e., between positions 28,510,120 and 33,480,577), raising the possibility that the SNP are in linkage with each other and with the HLA-B*57 haplotype.

Five SNP under-represented in LTNP, all mapping to chromosome 6, had a median allelic frequency of 0.10 (range 0.10–0.20) in the five HIC investigated, with 20% (range 20–40) of HIC carrying the minor allele in the SNP of interest. When the four Caucasian HIC were analyzed, the frequency of the minor allele was 0.13, with 25% of HIC carrying the minor allele in the SNP of interest.

The immunogenetics of elite control of HIV-1 infection in five patients revealed that HLA-B*5701, HLA-Cw*0602, HLA-DQ9, and multiple SNP present in or near MHC genes on chromosome 6 are strongly associated with elite control and that these alleles of the various MHC genes are present on an MHC ancestral haplotype.

## Discussion

The association between HLA-B*57 and -B*27 alleles and a slower rate of HIV-1 disease progression is strongly supported by host immunogenetics (7, 12, 18). HLA-B*57 is associated with protection from disease progression at early stages, epitomized by a delay in the HIV-1-induced CD4^+^ T-cell decline. HLA-B*57 was present in all the Caucasian HIC considered in our study and was greatly enriched compared to a control HIV-1-negative European population. In contrast, HLA-B*27, which is known to delay presentation of AIDS-defining illnesses once the CD4^+^ T-cell count drops below 200 cells/μl blood (19), was observed in 50% of Caucasian HIC studied. This temporal difference suggests that the anti-HIV-1 functions associated with these HLA types are distinct from one another. When both these alleles are present, as in two of the HIC in our study, the effects may be cumulative.

Natural killer (NK) cells exert a crucial antiviral function during the innate immune response against viral infections. The defined functional and phenotypic features of NK cells in HIC have underlined the contribution of innate immunity in control of viral replication (16). Recent studies have shown that NK cell subset distribution and function are affected by distinct levels of HLA-C (20). Hence, HLA-C expression levels by influencing both the innate and adaptive CD8 T-cell immunity impact both HIV-1 load and disease progression. We found that all five HIC in our study carried the HLA-Cw*0602 allele. HLA-Cw*0602 is an MHC class I allele that has been linked to enhanced HLA-expression and presentation of peptides (21). Additionally, it is associated with psoriasis, exhibits non-responsiveness to upregulation by key pro-inflammatory cytokines, including IFN-α (22), and is associated with delayed disease progression in HIV-1 infection (23). All four Caucasian HIC showed the HLA-B*57 and HLA-Cw*0602 allelic combination, which is also associated with HIV-1 non-progression (14). Moreover, HLA-Cw*6 has associations with the activating KIR 2DS1, the combination representing a major risk factor for psoriasis (24). HLA-C Cw*0602 has not been previously shown to have an independent effect in HIV-1 infection, although the interaction of its gene product with its appropriate cognate KIR molecule most probably plays a role. KIR-typing was not performed on these individuals; however, the interaction between MHC class I and KIR is likely to have implications in HIV-1 control and should be considered in future studies. Recent reports indicate that such studies are warranted which assess the consequences of distinct HLA-C expression, co-infections and, resulting interactions with KIR (20).

HLA-DQ9 was present in all Caucasian HIC and has also been associated with psoriasis (25), as well as other autoimmune disorders, including vitiligo (26) and juvenile diabetes (27). In generalized vitiligo, peripheral regulatory T cells exhibit impaired suppressive activity on autologous CD8 T cells (28), which react to self-peptide. A similar mechanism may operate in HLA-DQ9^+^ HIV-1-infected individuals, leading to increased activity of HIV-1-specific CD8 T cells, and favoring effective viral control.

Single-nucleotide polymorphisms identified from a large study of LTNP were present in a small cohort of HIC at a greatly enriched frequency and in combination with multiple other SNP of interest. The majority (22/24) of the SNP which were enriched in the HIC, map to the HLA region of chromosome 6, suggesting that HLA-Cw*0602, HLA-DQ9, and most of the SNP are in relatively strong genetic linkage with each other on the extended HLA-B*57 haplotype. Two of the SNP considered map on chromosome 8 and may not be in direct genetic linkage with HLA-B*57. However, we cannot exclude that even these two SNP may be in relatively strong linkage disequilibrium with the HLA region, irrespective of chromosomal separation. Thus, it is possible that all these markers represent the same single-genetic unit in the Caucasian HIC. Unfortunately, HLA type was not defined in LTNP from the GISHEAL and PRIMO cohorts, which prevented us from comparing the allelic frequencies of the SNP between the HIC in our study and a population of subjects carrying HLA-B*57 without the other variants. Consequently, we cannot determine whether the combination of HLA-B*57 with HLA-Cw*0602, HLA-DQ9, and the SNP is simply due to linkage disequilibrium or whether they represent multiple genetic factors with a cumulative beneficial effect on disease progression.

The rate of HIV-1 disease progression is a continuous variable (2), in that within a group of LTNP some individuals are able to control viral replication to higher or lower levels than others. Although HIC are exceedingly rare among HIV-1^+^ individuals, our data suggest that genetic profiles associated with non-progression may be observed at an even higher frequency in the subgroup of LTNP who, in addition to delayed disease progression, also control viral replication (1). As the HLA class I and II types of the patients had not been studied in the aforementioned GWAS study, the main new information reported is the class I and II types of the patients. Furthermore, the study describes the HLA associations of these exceptional HIC which show a strong enrichment of HLA-B*57 and B*27. Regardless of limitations, the goal was to demonstrate that extreme viral control, albeit rare, is associated with a strong genetic predisposition and that such genetic background is in turn a defining feature of HIC. Recognizing the processes behind this outermost long-term non-progressive phenotype is essential for gaining insight into the pathogenesis of HIV-1 disease and for the identification of an effective functional cure.

The group of patients studied is indeed unique emphasizing the value and rarity of the work presented herein, which results from the identification of HIC from more than 10 years of clinical follow-up, among one of the largest single center cohorts of people living with HIV in Europe. Findings imply a strictly defined group of HIC may be characterized by a “genetic signature,” representative of multiple determinants of resistance from disease progression. Future work into understanding the mechanisms behind the distinct immunological and virological outcome of these HIC profiles is required to develop potent immunotherapeutic strategies.

## Ethics Statement

This study was carried out in accordance with the recommendations of “NRES Directorate Guidelines, Riverside Research Ethics Committee” with written informed consent from all subjects. All subjects gave written informed consent in accordance with the Declaration of Helsinki. The protocol was approved by the “Riverside Research Ethics Committee.”

## Author Contributions

All authors conceived, designed, performed and analyzed the experiments, and contributed to the interpretation of results and writing of the manuscript. All authors contributed significantly to the work and have seen and agreed to all content in the manuscript, including the data as presented.

## Conflict of Interest Statement

The authors declare that the research was conducted in the absence of any commercial or financial relationships that could be construed as a potential conflict of interest.
